# The Annual Meeting of the Oldest Dental Society

**Published:** 1882-02

**Authors:** 


### THE ANNUAL MEETING OF THE OLDEST DENTAL SOCIETY.

Below is given the programme of the next Annual Meeting of Mississippi Valley Dental Society, the oldest existing body of the kind in the world. It was organized at a time when there was but two or three similar bodies extant, and these have long since been suspended. For many years after its organization, its members were from the West as far as St. Louis, from the South as far as Memphis and Nashville, from Pittsburgh on the East and from Buffalo and Chicago on the North.

It embraced during the first ten to fifteen years of its career most of the prominent dentists of the territory here indicated. The membership became more circumscribed as other societies were formed, so that now its membership is small compared with that of former times. But the interest in it has always been well sustained, especially by the older members, who have a generous satisfaction in what has been accomplished by it. And not the least of that which it accomplished was the training of its members for efficient work in other similar bodies. Just how much has been done in this direction, it is impossible to estimate, but it is indisputable, that many of our societies throughout the country have to a greater or less extent derived benefits from this source.

It is not to be doubted for a moment, that the approaching meeting will be equal to those of former years; and, indeed, why shall it not be better, and it will, if all who should be are present. Then let all come who can.

